# On the Pulsating Tumours of Bone; with the Account of a Case in Which a Ligature Was Placed around the Common Iliac Artery

**Published:** 1845-05

**Authors:** Edward Stanley

**Affiliations:** Surgeon to St. Bartholomew’s Hospital


ROYAL MEDICAL AND CHIRURGICAL SOCIETY, MARCH 11, 1845.

*On the Pulsating Tumours of bone; with the account of a case in which a Ligature was placed around the Common Iliac Artery.* By EDWARD STANLEY, F. R. S., Surgeon to St. Bartholomew's Hospital.—The author remarks that there are three distinct sources of pulsation in the tumours of bone. 1st, the proximity of the tumour to a large artery—2nd, the developement of blood-vessels and blood-cells, constituting a sort of erectile tissue within the tumour—3rd, the enlargement of the arteries of the bone in which the tumour has arisen. Proximity to a large artery is the most frequent source of pulsation in these tumours, of which six examples are brought forward. Three occurred at St. Bartholomew's Hospital. In one of them, an encephaloid tumour originating in the humerus, the ligature of the subclavian was recommended, but not assented to by the patient. In another, it was agreed in consultation that sufficient ground existed for believing the tumour to be a popliteal aneurism, and, accordingly, the femoral artery was tied in the middle of the thigh. The tumour consisted of a compound of soft fibrous and dense osseous tissue, the latter situated deeply, and extending around the femur, in which it appeared to have originated. Of the other three cases, two were communicated by Mr. Hodgson, of Birmingham, and the third by Mr Lawrence. The latter is already recorded in the 17th volume of the transactions of the Society. There are six examples of pulsating tumours, differing in their nature, and originating in different bones, but agreeing in the circumstance, that no other source of pulsation was discoverable in them than the contiguity of large arteries. To the same class of cases, the author considers that the important one recorded by Mr. Guthrie belongs, in which a medullary tumour, about as large as an adult head, situated upon the right nates of a female, presented so decidedly the characters of aneurism, that it was believed to be so by Sir Astley Cooper, and other experienced surgeons who were consulted upon the case, and, accordingly, a ligature was placed around the common iliac artery.

On the subject of pulsation in the tumours of bone, dependent on the developement of blood-vessels and blood-cells forming a sort of erectile tissue within the tumour, Mr. Stanley remarks, "that in the case of recent occurrence in St. Bartholomew's Hospital, there certainly was a structure capable of enlargement by the distention of its vessels and cells, and assuming these to have been continuous with the surrounding arteries, the rush of blood into this structure might give the whole mass a pulsation resembling that of aneurism. Two cases are related, in which the pulsation of the tumour was ascribed to a similar cause. In one, communicated to the author by Mr. John Lawrence, junior, the tumour, originating in the upper part of the femur, was more of a gelatinous than encephaloid character, and its vascular tissue formed more than one half its bulk. The other, which was furnished by Mr. Luke, of the London Hospital, was a tumour of the lower part of the femur, and in consequence of suspicion of aneurism, the femoral artery was tied. The limb was subsequently amputated, when the tumour was found to consist of cells of varying size, some of the largest being about an inch in diameter, and they were filled with blood.

Of pulsation in the tumour of bone dependent on the enlargement of the arteries of the osseous tissue, several cases are referred to; one related by Dupuytren; the others by Pelletan.

One circumstance in the history of these different forms of pulsating tumour is especially noticed by the author, as it appears to have a material influence on the production of pulsation in them; this is, the density and resistance derived in one or other direction from the bone or its coverings. A tumour originating in soft parts, and unconnected with any bone, but situated close to a large artery, and confined within resisting structures, and thus approximating in its conditions to the pulsating tumour of bone, may, like it, pulsate in a manner to be mistaken for aneurism. An example is given of a man admitted into St. Bartholomew's Hospital, under the care of Mr. Earle, with a pulsating tumour below the left clavicle, which presented all the characters of aneurism, and, accordingly, a ligature was placed around the subclavian artery. The tumour subsided sufficiently to confirm the opinion entertained of its nature, and the patient was discharged; six years afterwards he was again admitted with general derangement of the health, from which he sunk. On dejection, the axillary artery presented no indication of having been the seat of aneurism. Immediately behind the artery was a solid tumour, which had originated within the sheath of a large nerve.

After some observations, tending to show the little value to be attached to the presence of bellows' sound in the diagnosis between aneurism and the pulsating tumour of bone, the author proceeds to relate the case of pulsating tumour of the ilium which has recently occurred in St. Bartholomew's Hospital, in which a ligature was placed around the common iliac artery. The patient, a man aged forty-two, had, on the inner side of the right upper arm, a tumour about the size of a small orange, very loosely connected with the surrounding structures, free from pain, and without pulsation. This tumour was first observed about ten years ago, and during the last three years it had ceased to grow. The pulsating tumour of the pelvis had its chief attachment to the left ilium, and projected from both surfaces of the bone. It reached downward to Poupart's ligament, and to the extent of about three inches into the abdomen. It felt moderately firm, and a little below the crista, near the anterior superior spine, a small movable piece of bone was discovered, apparently involved in the tumour. Everywhere within the reach of the fingers, the tumour pulsated, not with a thrill or vibration, but with the deep heavy beat of aneurism. By the ear resting against the abdominal parietes, a bellows' sound was plainly recognised. After a minute description of the local features and constitutional phenomena of the disease, the author observes, that in deciding upon the nature and treatment of the case, the following points were involved:—was this pulsating tumour an aneurism? and if so, from what artery had it arisen? or was it one of the pulsating tumours of bone? He then states the arguments which, in consultation, led to a preponderance of opinion in favour of this tumour being an aneurism. In the uncertainty respecting the origin of the supposed aneurism from the external or internal iliac artery, the decision would obviously be that the common iliac should be tied, and the man having decidedly expressed his feeling in favour of submitting to the operation, the author considered it his duty to undertake it. The operation was performed on Monday, 27th January. The case proceeded favourably to the middle of the second day, when symptoms of peritonitis ensued, and he sank on the morning of the third day from the operation. On examining the body, the effects of peritonitis were observed in the deeper parts and left side of the abdomen. In the wall of the left ventricle of the heart there was a medullary tumour about the size of a filbert. Medullary matter was found in the bronchial glands, and a few deposites of the same kind in the lungs. A minute description is given of the tumour in the pelvis, which was connected with the ilium, and composed of a spongy tissue, with cells and convoluted vessels distributed through it. The tumour in the arm, which had all the marks of an innocent structure, was found, to the surprise of the author, identical in structure with the tumour in the pelvis,

This paper is concluded by some remarks on the operation of tying the common iliac, or internal iliac artery, or the external iliac near its origin, tending to show that with the least risk of injury to the peritoneum, the readiest mode of reaching these vessels must be by an incision through the posterior part of the abdominal parietes.

Mr. Toynbee briefly referred to a case he had lately dissected of a young man who had died of consumption at the age of 19. The patient had a pulsating tumour of bone differing from Mr. Stanley's cases, inasmuch as it was composed almost entirely of bloodvessels, and contained no cells or other structure. It was situated at the point of ossification of the parietal bones.

Mr. Fergusson regarded the paper of Mr. Stanley with much interest. It showed the great difficulty which experienced surgeons might encounter in deciding on the true nature of certain kinds of tumours connected with the pelvic region. He had seen several cases similar to those brought under notice by the paper. Two of these cases had been under the care of Mr. Syme, of Edinburgh, and illustrated the difficulty of diagnosis of these cases, even to experienced and able surgeons. These cases occurred during his, Mr. Fergusson's, connexion with the Royal Infirmary, and he had consequently had an opportunity of seeing them. Mr. Syme had published the cases in his Clinical Reports. In one of these cases there was a swelling in the situation of the external iliac artery, which was considered to be aneurismal; other surgeons, however, were of a different opinion. Mr. Syme, however, acting chiefly on his own responsibility, determined on placing a ligature high up on the external iliac, or on the common iliac artery, as might seem advisable. On completing the incisions through the abdominal parietes, it was discovered that the tumour was not an aneurism; it was nevertheless removed, and the patient sunk a few days after the operation. It was then discovered that tumours of a similar kind were situated in various parts along the course of the chief arterial vessels; they were smaller in size than that removed; but it was evident that they owed any pulsation they possessed to their connexion with the arteries. In a second case, in which a tumour was also situated in the course of the external iliac, much difference of opinion existed amongst the most distinguished surgeons of Edinburgh, respecting its true nature. Eventually a ligature was placed round the common iliac artery. The patient died, and the tumour was found to be aneurismal. Another case which had come under the observation of the speaker, resembled, in many points, the cases mentioned by Mr. Stanley. The pulsation and the bruits, said to be diagnostic of aneurism, were present; he, however, refused to operate. He was led to this determination from having become recently acquainted with Mr. Guthrie's case, to which reference had been made in the paper. After death the tumour was found to be exceedingly similar to the cases mentioned in the paper. The tumour was prominent in the pelvis, both internally and externally, and the os innominatum was completely destroyed. The tumour was composed chiefly of medullary matter, clots of blood, and bony spiculæ. Mr. Nicol, of Inverness, about the time of the occurrence of these cases, had treated a tumour connected with the upper part of the shoulderbone, supposing it to be aneurismal. The case was similar to one related in the paper by Mr. Luke, inasmuch as the tumour had resulted from an injury. A ligature was placed round the subclavian. At first, the operation appeared successful, but the patient soon died, and the tumour was found to be malignant.—*London Lancet.*

